# Supplementary material for: TMEM16 scramblases thin the membrane to enable lipid scrambling
Source: Nat Commun. 2022 May 11;13:2604. doi: 10.1038/s41467-022-30300-z (PMC9095706; doi:10.1038/s41467-022-30300-z)
Supplement: Supplementary file 1 — Supplementary Information [file 41467_2022_30300_MOESM1_ESM.pdf]

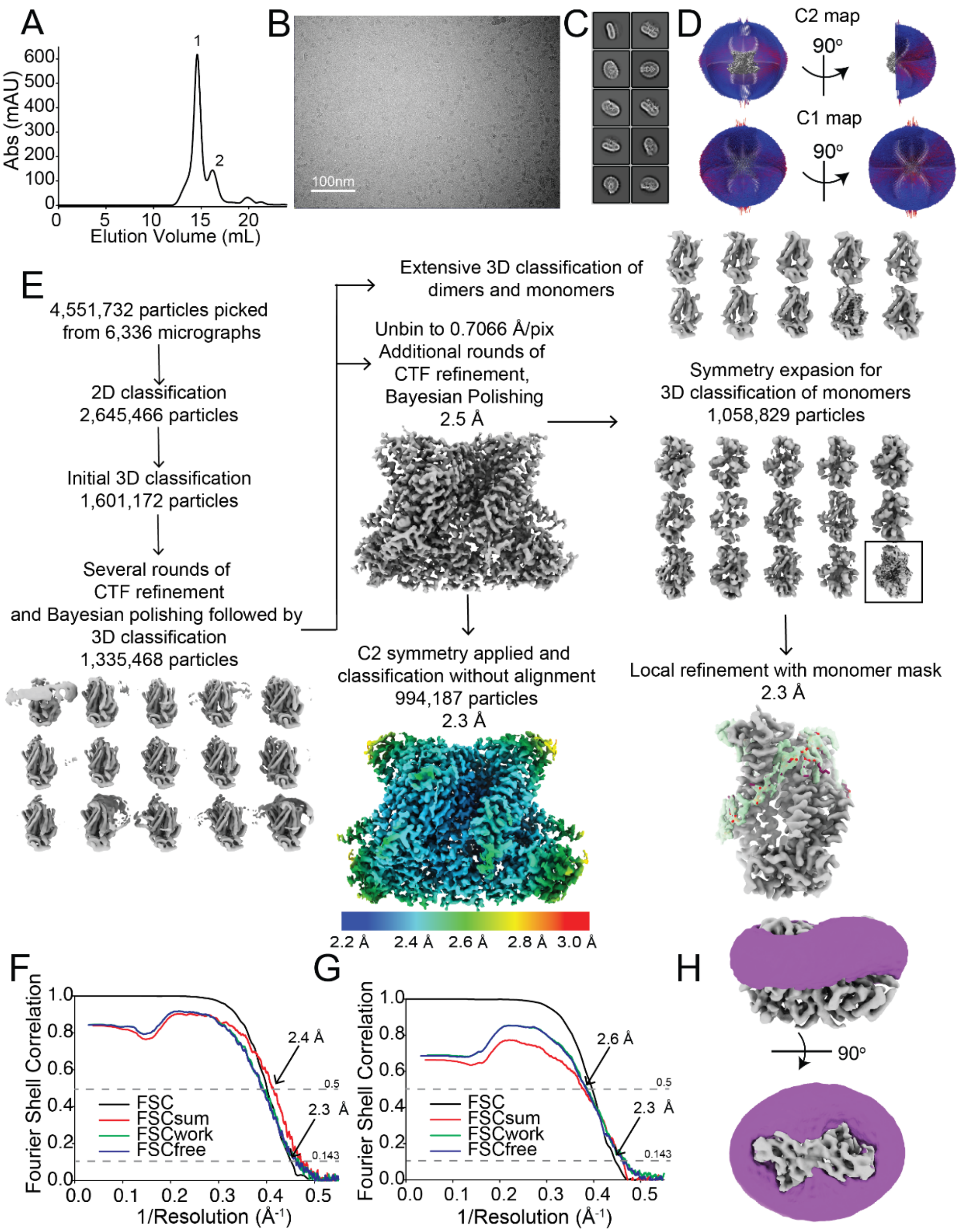

Supplementary Figure 1: **Structural determination of afTMEM16 in C18 lipids in the presence of Ca^{2+}.** A: Size exclusion profile of afTMEM16 reconstituted into nanodiscs containing MSP1E3 scaffold protein and C18 lipids in the presence of Ca^{2+}. 1 indicates the main peak and 2 the empty nanodiscs. B: representative micrograph. C: representative 2D classes of afTMEM16/nanodisc complex. D: Angular distribution of final C2 (top) and C1 (bottom) reconstructions. E: Image processing workflow including symmetry expansion and classification to identify monomer class with well-defined lipids used for building. F-G: FSC plots for dimeric afTMEM16/nanodisc complex. Final masked reconstruction colored by local resolution calculated using the Relion implementation. (F) and monomer class with resolved lipids (G). FSC is between the two half maps to determine the resolution of the reconstruction evaluated at 0.143 cutoff. FSCsum, FSCwork, and FSCfree are model validations evaluated at 0.5 cutoff (see methods). H: Architecture of the nanodisc surrounding afTMEM16. The final reconstruction was filtered to 7 Å. The nanodisc density is shown in purple and the protein is shown in grey.

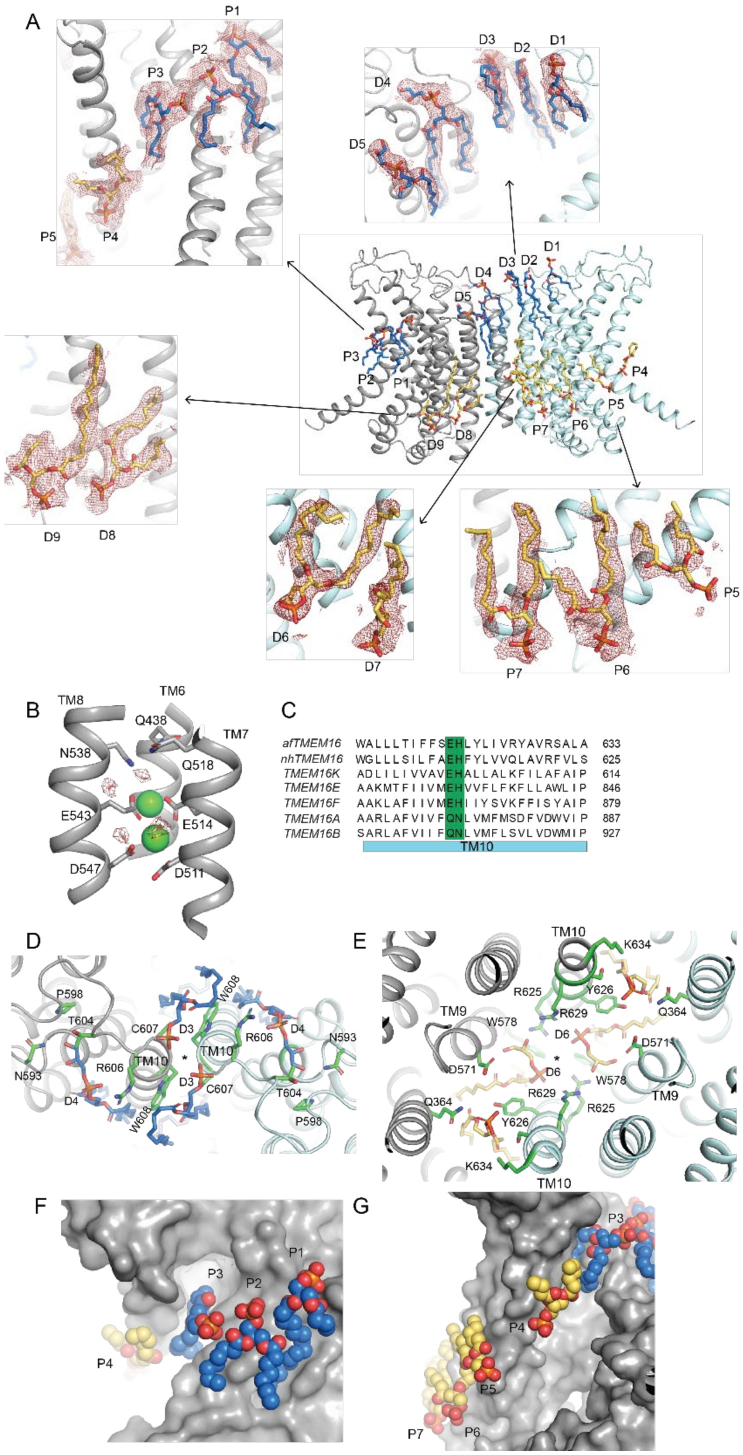

Supplementary Figure 2: Ligand densities associated with afTMEM16 in the C18/Ca^{2+} structure. A: Mesh of modeled lipids in the afTMEM16 C18/Ca^{2+} structure. Lipids are shown in blue (outer leaflet) and yellow (inner leaflet) with mesh from the unsharpened map in red with σ=5.5. B: Water molecules coordinating the two Ca^{2+} ions. Mesh from the final map is shown in red at σ=8. For clarity, E445 and G441 are not shown. C: Structure-based sequence alignment of TM10 showing the conservation of the dimer interface salt bridge. The alignment was generated using PROMALS3D (Pei and Grishin, 2014). D-E: Coordination of lipids associated with the dimer interface at the outer (D) and inner (E) leaflets. Lipids are shown as blue (outer leaflet) or yellow (inner leaflet) sticks and colored in CPK. afTMEM16 monomers are in gray and cyan. Coordinating side chains are shown as green sticks and colored by CPK. F-G: View of pathway lipids and their tails intercalated with interhelical cavities. afTMEM16 is shown as surface and lipids are shown in spheres colored as in D-E.

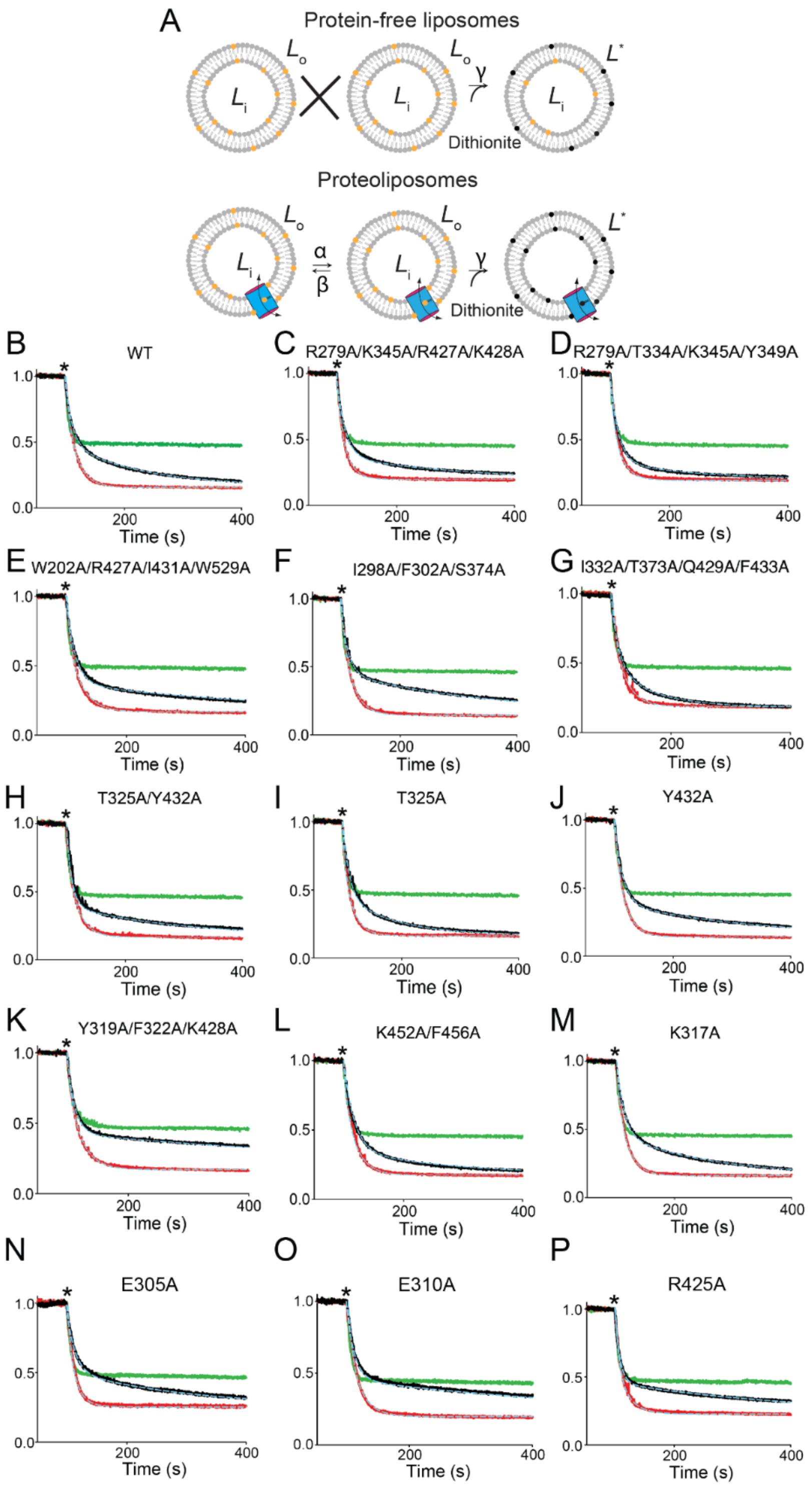

Supplementary Figure 3: **Representative traces of scrambling experiments of WT and mutant afTMEM16.** A: Schematic of the in vitro scramblase assay. Liposomes are reconstituted with NBD-labeled phospholipids (orange) that distribute equally in the two leaflets. Addition of extraliposomal sodium dithionite reduces the NBD fluorophore (black), causing 50% fluorescence loss in protein-free vesicles (top panel). When a scramblase is present (bottom panel), all NBD-phospholipids become exposed to dithionite, resulting in complete loss of fluorescence. B-P: Representative time course of scrambling experiments of wildtype (B) and of indicated afTMEM16 mutants implicated in lipid coordination outside the permeation pathway (C-E), or located in the extracellular vestibule or central constriction (F-P). * denotes addition of sodium dithionite. Traces: protein-free (green), afTMEM16 proteoliposomes +0.5 mM Ca^{2+} (red) or 0 Ca^{2+} (black), fits to Eq. 1 (dashed cyan).

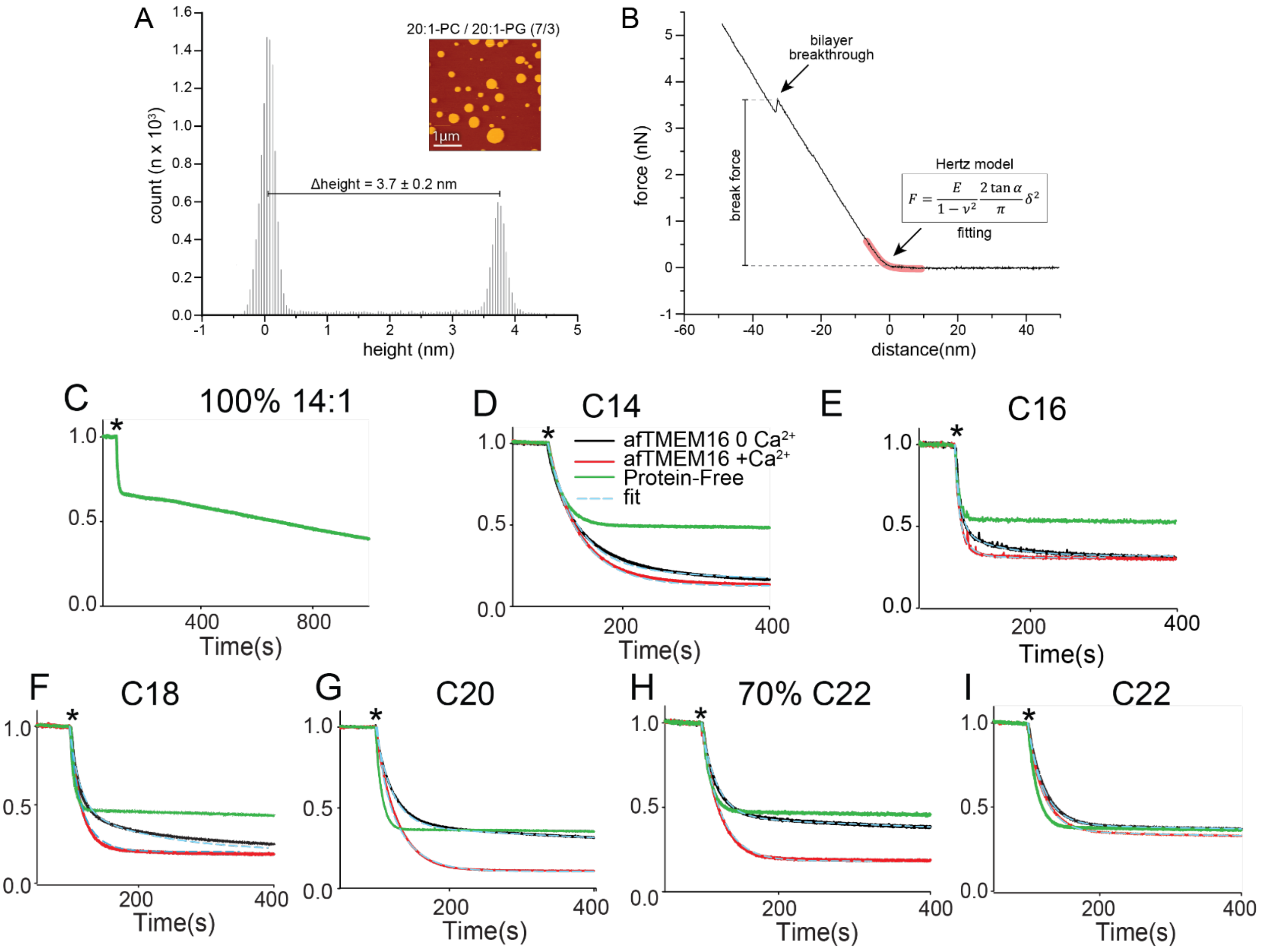

Supplementary Figure 4: **Lipid bilayer characterization by atomic force microscopy and functional modulation of afTMEM16 by bilayer thickness.** (A) Pixel height histogram of (70% 20:1 PC/30% 20:1 PG) membranes based on AFM topography images (inset). The mica level is set to 0.0 nm and the height difference to the distribution of pixel heights from the membrane patched is measured Δh = 3.7±0.2 nm (Peak ± FWHH of the value distribution). (B) The mechanical properties (Young's modulus and breakthrough force) of the membrane characterized in a force-distance curve. The Young's modulus is derived by fitting the approaching part of force-distance curve with Hertz model (red curve). The break force is characterized by a ~3 nm cantilever relaxation at ~3.5nN force (in this case). C: Time course of protein-free liposomes formed from 14:1 lipids alone. D-I: time course of scrambling by afTMEM16 in C14 lipids (D), C16 lipids (E), C18 lipids (F), C20 lipids (G), 70% C22 lipids (H) and C22 lipids (I). * denotes addition of sodium dithionite. Traces: protein-free (green), afTMEM16 proteoliposomes +0.5 mM Ca^{2+} (red) or 0 Ca^{2+} (black), fits to Eq. 1 (dashed cyan). Panel F and I are alternate repeats of results from a previous study {Falzone, 2019 #2993}.

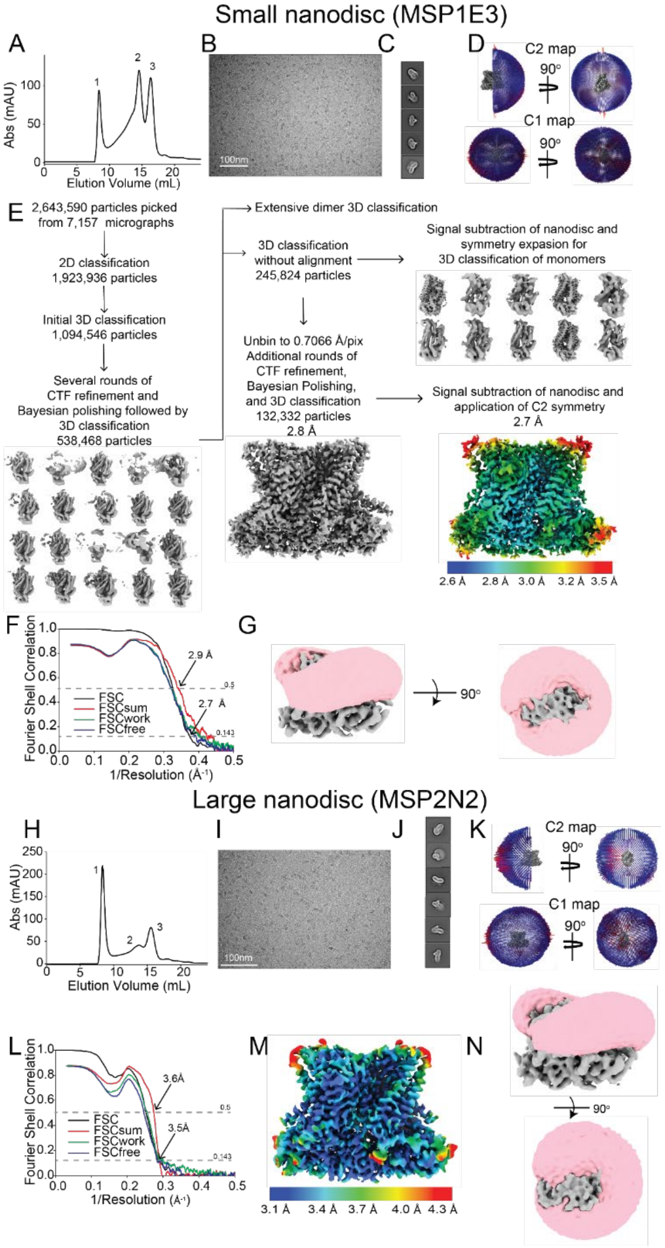

Supplementary Figure 5: **Structural determination of afTMEM16 in C22 lipids in MSP1E3 and MSP2N2 nanodiscs in 0.5 mM Ca^{2+}.** A: Size exclusion profile of afTMEM16 reconstituted into nanodiscs containing MSP1E3 scaffold protein and C22 lipids in 0.5 mM Ca^{2+}. 1 indicates void peak, 2 the main peak and 3 empty nanodiscs. B: representative micrograph. C: representative 2D classes of afTMEM16/nanodisc complex. D: Angular distribution of final C2 (top) and C1 (bottom) reconstructions. E: Image processing workflow including signal subtraction of the nanodisc density to apply two-fold symmetry. Final masked reconstruction colored by local resolution calculated using the Relion implementation.. F: FSC plots for afTMEM16/nanodisc complex. FSC is between the two half maps to determine the resolution of the reconstruction. FSCsum, FSCwork, and FSCfree are model validations (see Methods). G: Architecture of the nanodisc surrounding afTMEM16. The final reconstruction was filtered to 7 Å. The nanodisc density is shown in pink and the protein is shown in grey. H: Size exclusion profile of afTMEM16 reconstituted into nanodiscs containing MSP2N2 scaffold protein and C22 lipids in the presence of Ca^{2+}. 1 refers to the void peak, 2 refers to the main peak and 3 refers to empty nanodiscs. I: representative micrograph. J: representative 2D classes of afTMEM16/nanodisc complex. K: Angular distribution of final C2 (top) and C1 (bottom) reconstructions. L: FSC plots for afTMEM16/nanodisc complex. FSC is between the two half maps to determine the resolution of the reconstruction evaluated at 0.143 cutoff. FSCsum, FSCwork, and FSCfree are model validations evaluated at 0.5 cutoff (see Methods). M: Final masked reconstruction colored by local resolution calculated using the Relion implementation. N: Architecture of the nanodisc surrounding afTMEM16. The final reconstruction was filtered to 7 Å. The nanodisc density is shown in pink and the protein is shown in grey.

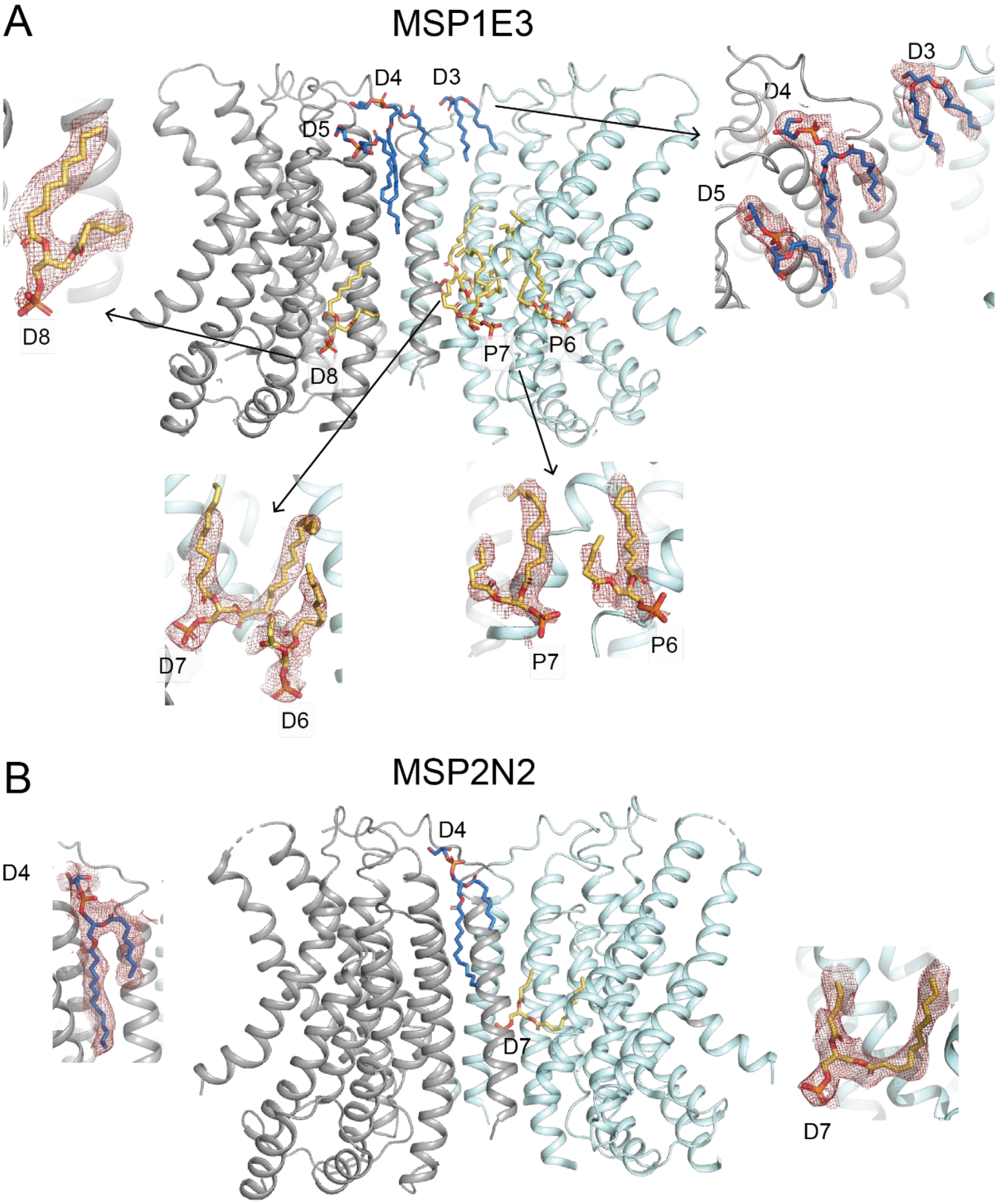

Supplementary Figure 6: Lipid densities associated with afTMEM16 in the C22/Ca^{2+} structures. A-B: Lipid densities associated with Ca^{2+}-bound afTEMEM16 in final unsharpened map from MSP1E3 nanodiscs (A) or from local resolution-filtered map of MSP2N2 nanodiscs (B). Upper and inner leaflet lipids are respectively shown as blue or yellow sticks. All lipids are labeled according to the naming in the C18/Ca^{2+} structure (Fig. 1). Density is shown in red at σ=5.5.

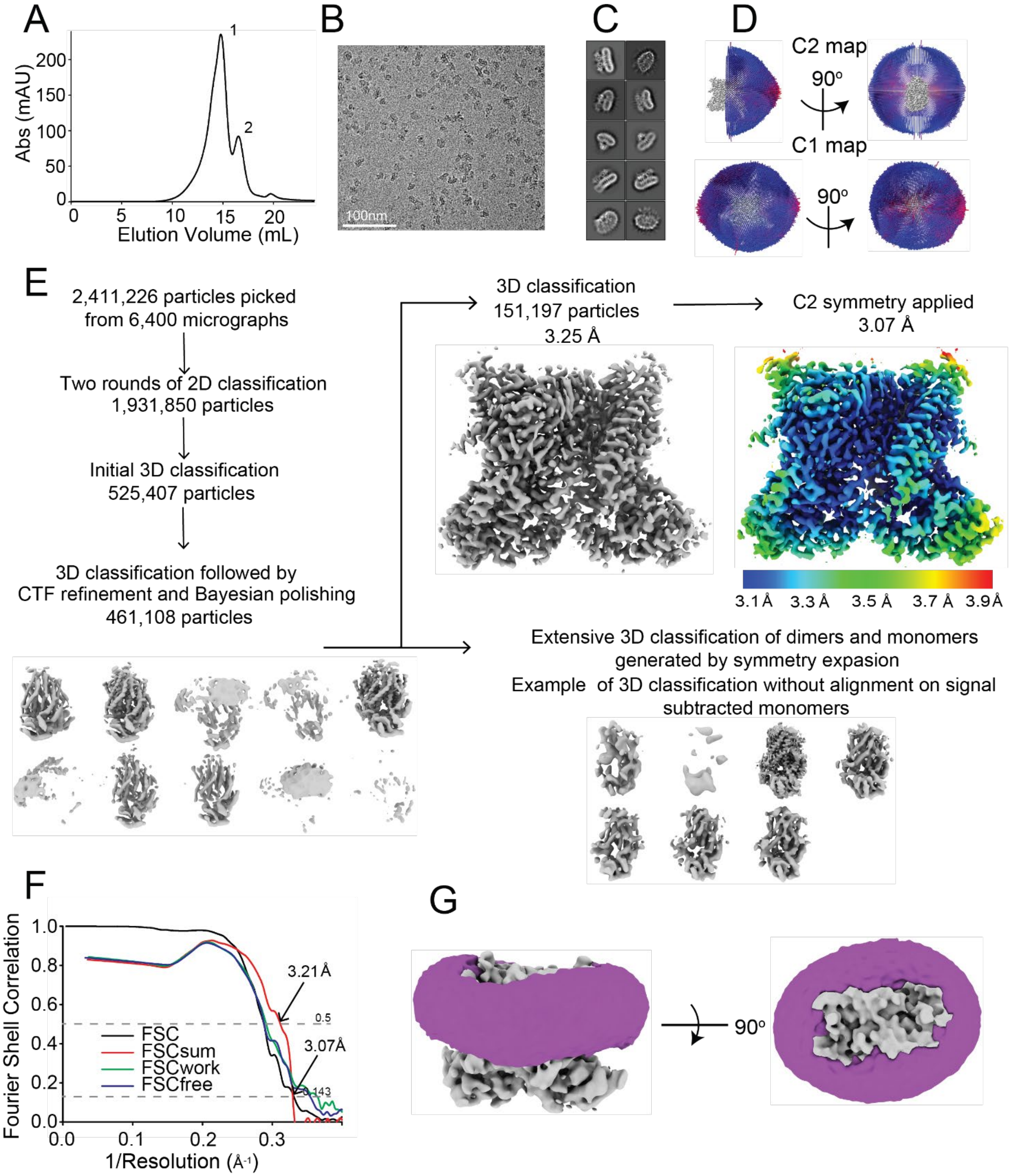

Supplementary Figure 7: Structural determination of afTMEM16 in C18 lipids in MSP1E3 nanodiscs in 0 Ca^{2+}. A: Size exclusion profile of afTMEM16 reconstituted into nanodiscs containing MSP1E3 scaffold protein and C18 lipids in the absence of Ca^{2+}. 1 indicates the main peak and 2 the empty nanodiscs peak. B: Representative micrograph. C: Representative 2D classes of afTMEM16/nanodisc complex. D: Angular distribution of final C2 (top) and C1 (bottom) reconstructions. E: Image processing workflow. Final masked reconstruction colored by local resolution calculated using the Relion implementation. F: FSC plots for afTMEM16/nanodisc complex. FSC is between the two half maps to determine the resolution of the reconstruction. FSCsum, FSCwork, and FSCfree are model validations (see methods). G: Architecture of the nanodisc surrounding afTMEM16. The final reconstruction was filtered to 7 Å. The nanodisc density is shown in purple and the protein is shown in grey.

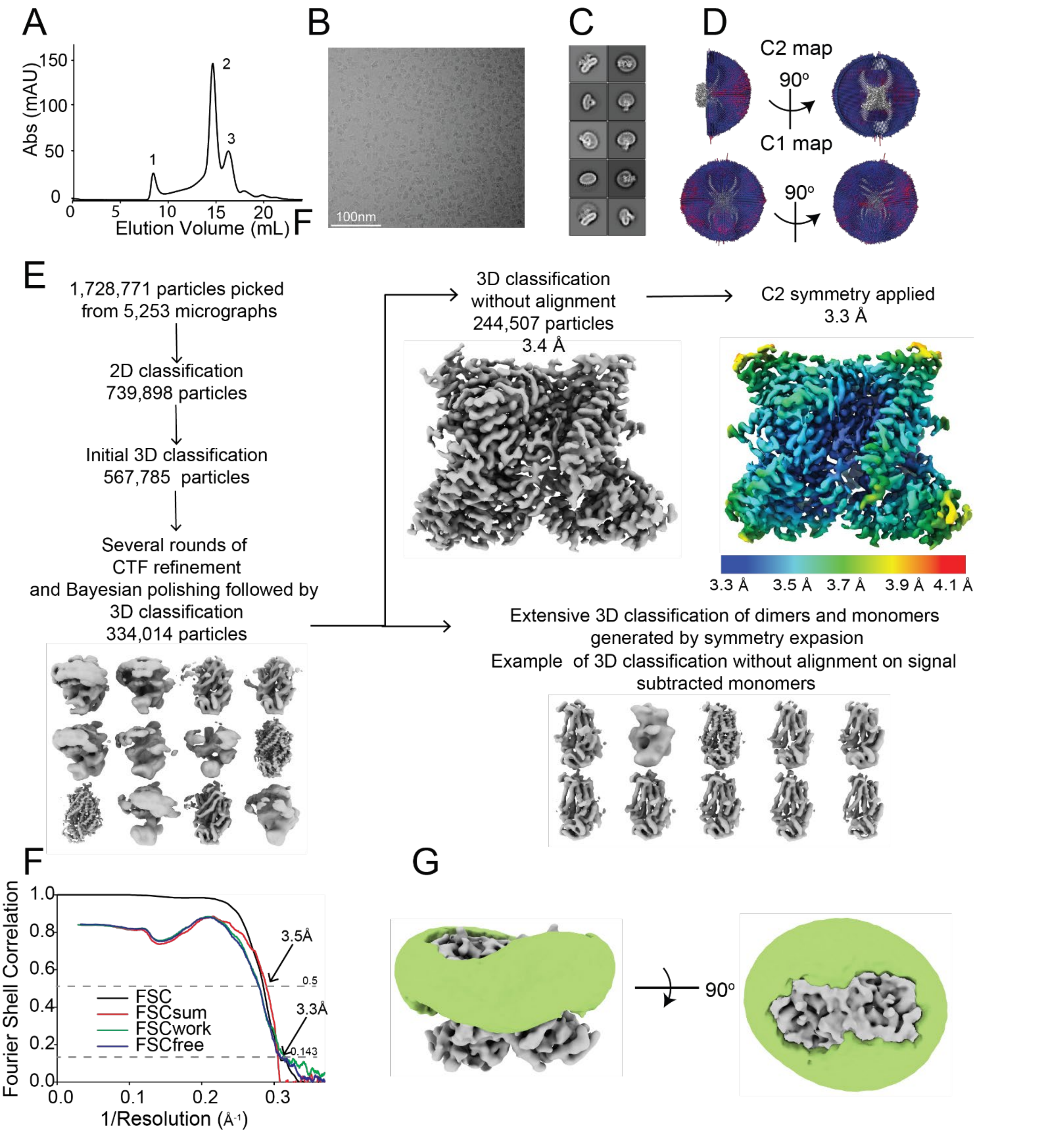

Supplementary Figure 8: **Structural determination of afTMEM16 in C14 lipids in MSP1E3 nanodiscs in 0 Ca^{2+}.** A: Size exclusion profile of afTMEM16 reconstituted into nanodiscs containing MSP1E3 scaffold protein and C14 lipids in 0 Ca^{2+}. 1 indicates void peak, 2 the main peak and 3 empty nanodiscs peak. B: Representative micrograph. C: Representative 2D classes of afTMEM16/nanodisc complex. D: Angular distribution of final C2 (top) and C1 (bottom) reconstructions. E: Image processing workflow. Final masked reconstruction colored by local resolution calculated using the Relion implementation. F: FSC plots for afTMEM16/nanodisc complex. FSC is between the two half maps to determine the resolution of the reconstruction. FSCsum, FSCwork, and FSCfree are model validations (see methods). G: Architecture of the nanodisc surrounding afTMEM16. The final reconstruction was filtered to 7 Å. The nanodisc density is shown in green and the protein is shown in grey.

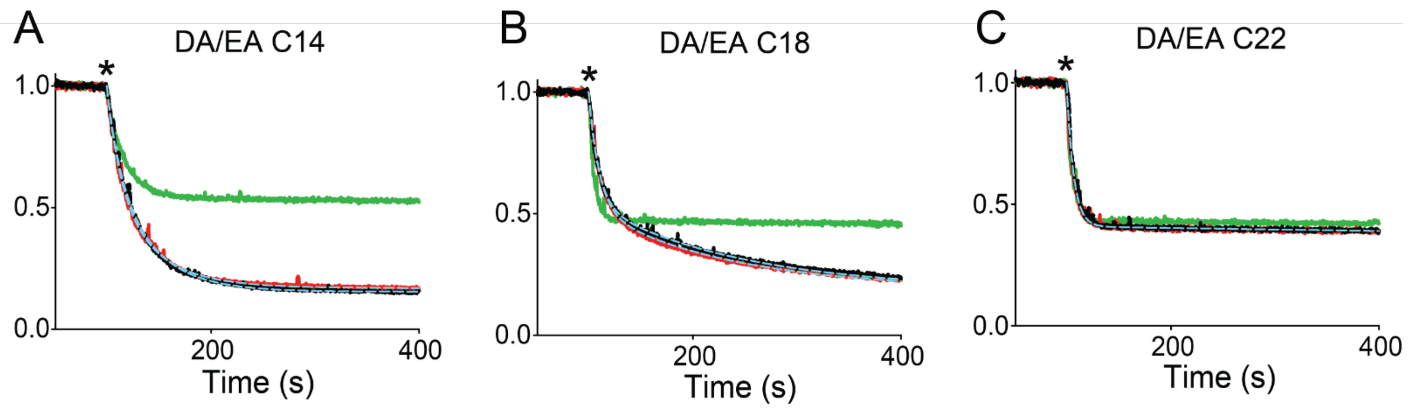

Supplementary Figure 9: **Representative traces of scrambling experiments of afTMEM16 D511A/E514A in membranes of different thickness.** A-C: Time course of scrambling by afTMEM16 D511A/E514A in C14 lipids (A), C18 lipids (B), C22 lipids (C). * denotes addition of sodium dithionite. Traces: protein-free (green), afTMEM16 proteoliposomes +0.5 mM Ca^{2+} (red) or 0 Ca^{2+} (black), fits to Eq. 1 (dashed cyan).

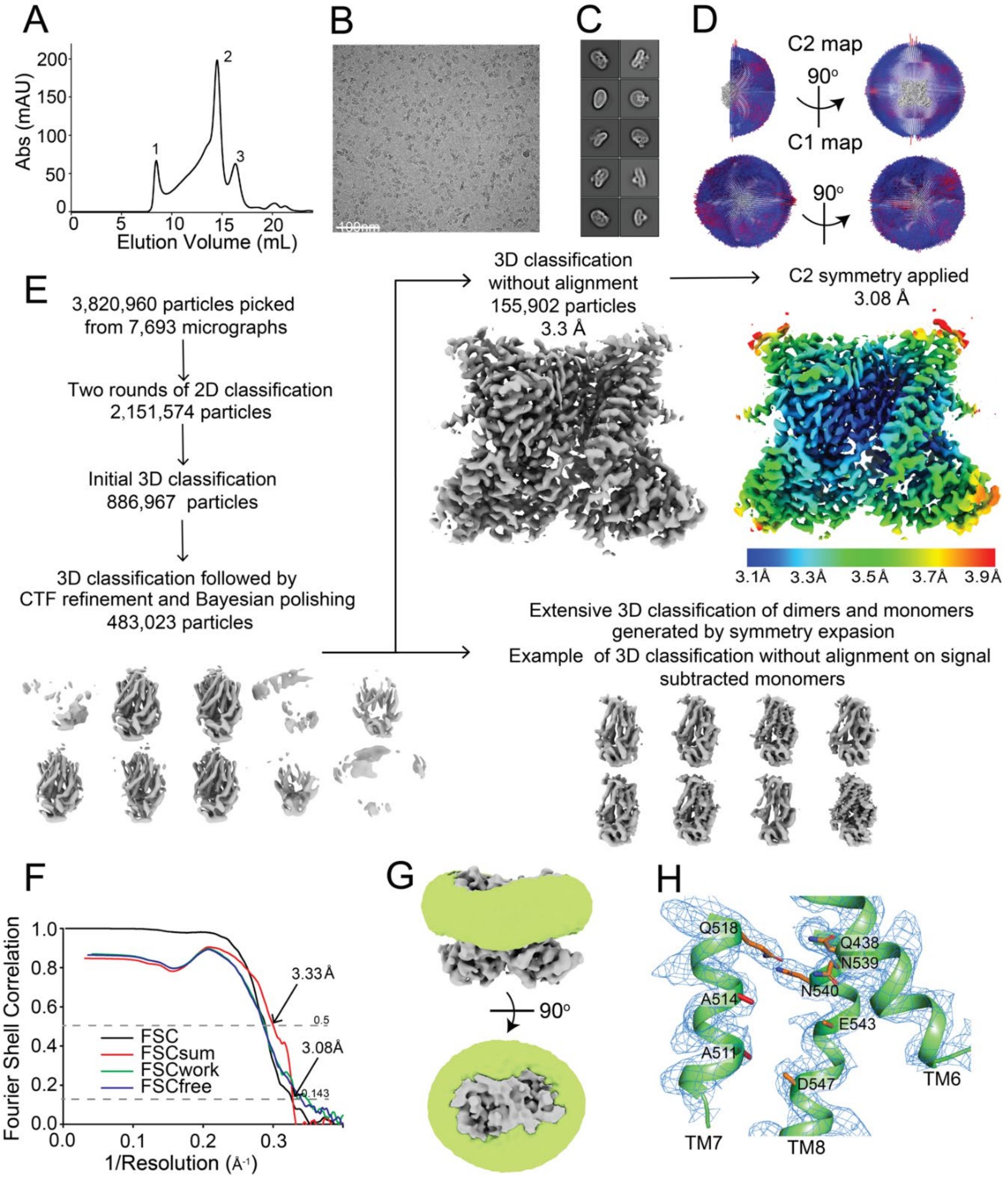

Supplementary Figure 10: **Structural determination of afTMEM16 D511A/E514A in C14 lipids in MSP1E3 nanodiscs in 0.5 mM Ca^{2+}.** A: Size exclusion profile of afTMEM16 D511A/E514A reconstituted into nanodiscs containing MSP1E3 scaffold protein and C14 lipids in the presence of 0.5 mM Ca^{2+}. 1 indicates the void peak, 2 the main peak and 3 empty nanodiscs peak. B: Representative micrograph. C: Representative 2D classes of afTMEM16/nanodisc complex. D: Angular distribution of final C2 (top) and C1 (bottom) reconstructions. E: Image processing workflow. Final masked reconstruction colored by local resolution calculated using the Relion implementation. F: FSC plots for afTMEM16/nanodisc complex. FSC is between the two half maps to determine the resolution of the reconstruction. FSCsum, FSCwork, and FSCfree are model validations (see methods). G: Architecture of the nanodisc surrounding afTMEM16. The final reconstruction was filtered to 7 Å. The nanodisc density is shown in green and the protein is shown in grey. H: Density of Ca^{2+} binding site. Side chains of putative Ca^{2+} binding residues in on TM6-8 are displayed as sticks except E543 and D547 due to their weak side chain densities. The two mutated residues A514 and A511 are highlighted in red. Density is shown in blue at σ=5.

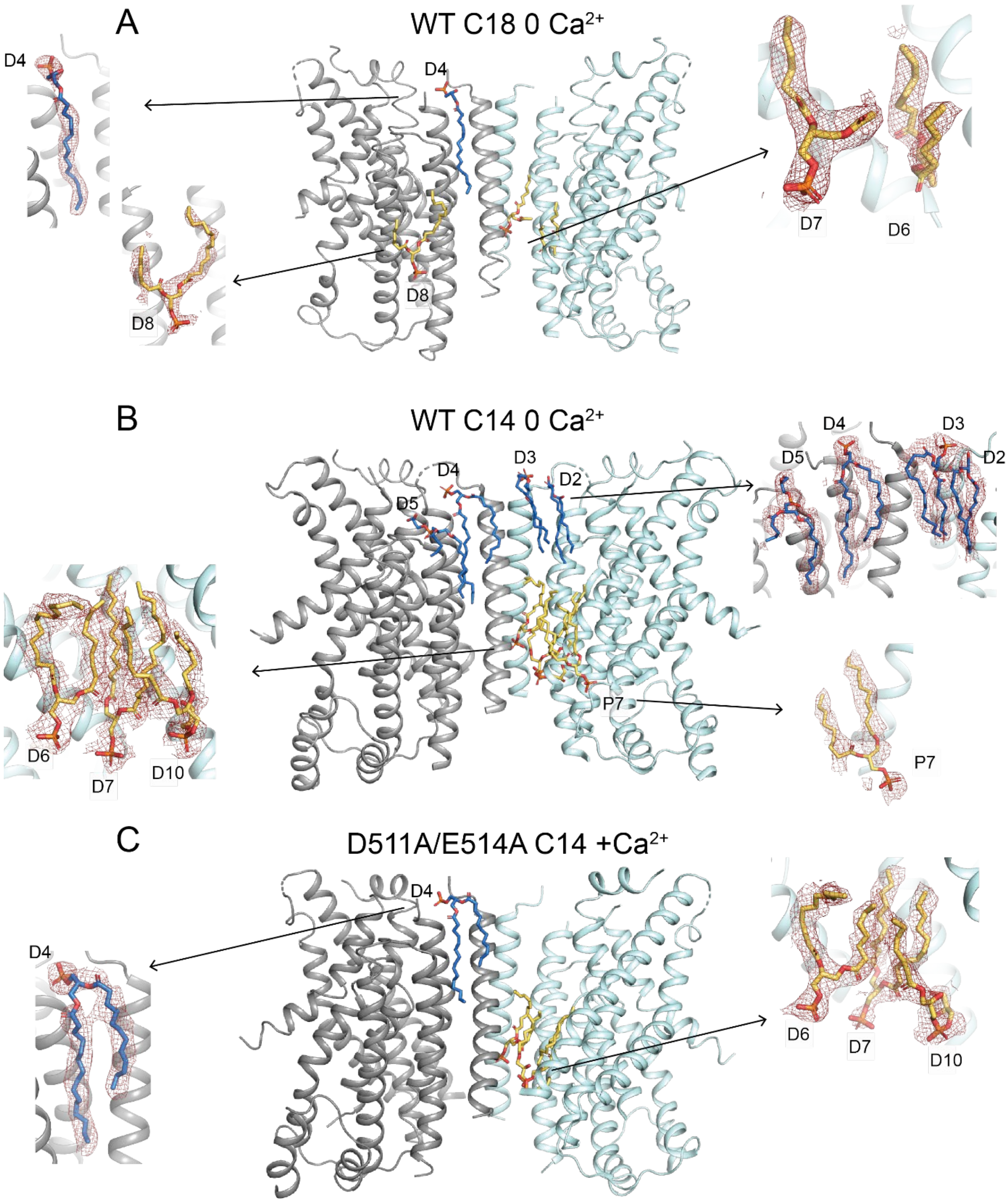

Supplementary Figure 11: Lipid densities associated with afTMEM16 in the apo structures. A-C: Lipid densities from the final maps associated with WT afTEMEM16 in MSP1E3 nanodiscs formed from C18/0 Ca^{2+} (A), C14/0 Ca^{2+} (B) and D511A/E514A afTMEM16 in C14/+Ca^{2+} (c). Upper and inner leaflet lipids are respectively shown as blue or yellow sticks. Lipids are labeled according to the naming in the C18/Ca^{2+} structure. D10 is resolved only in the WT C14/ 0Ca^{2+} and D511A/E514A afTMEM16 in C14/+$Ca^{2+.}$ Density is shown in red at σ=5.

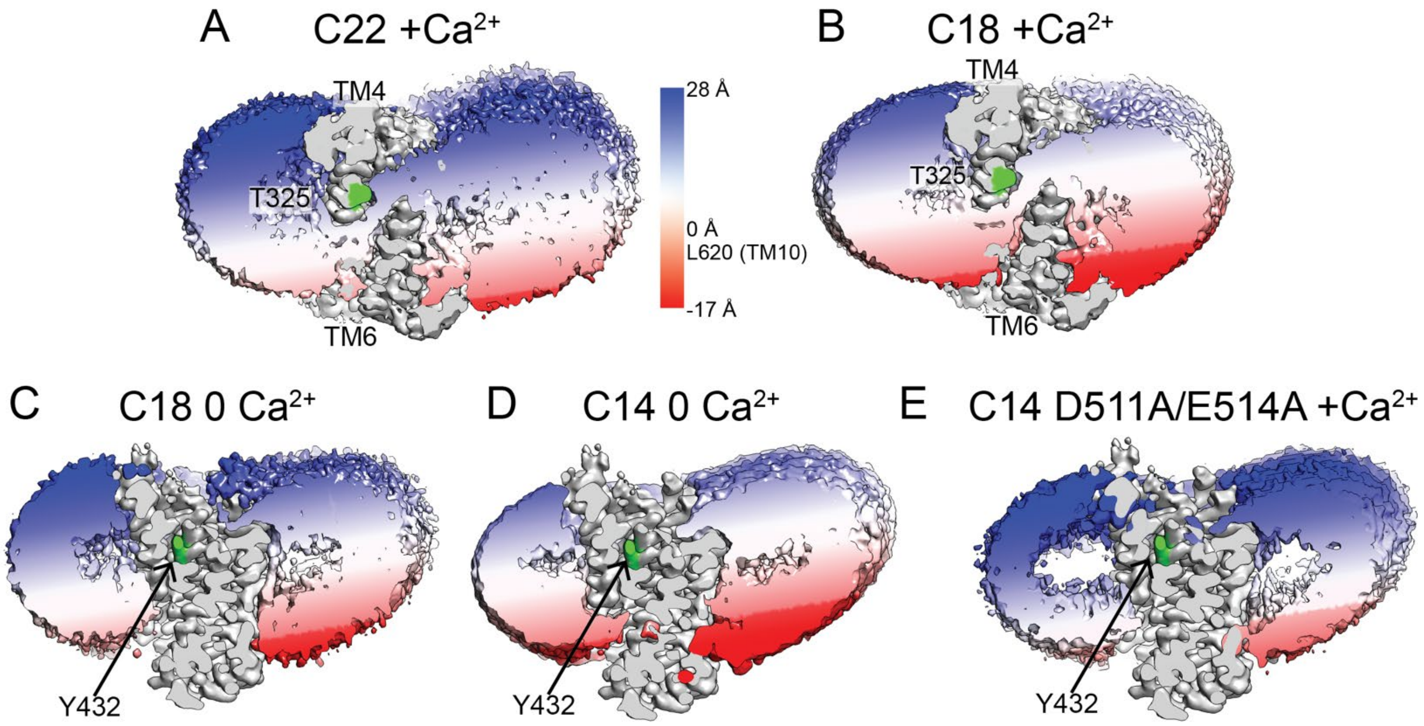

Supplementary Figure 12: Acyl chain length-dependent membrane thinning at the permeation pathway of afTMEM16. A-E Cutaway view of the segmented nanodisc density map near the pathway is viewed from the plane of the membrane for WT afTMEM16 in C22/Ca^{2+} (A), C18/Ca^{2+} (B), C18/0 Ca^{2+} (C), C14/0 Ca^{2+} (D), and C14/Ca^{2+} for the D511A/E514A mutant. The C1 final unsharpened maps containing nanodisc densities were aligned, resampled on the same grid, and colored according to the Z coordinate using UCSF Chimera. The density corresponding to the protein is shown in gray. Nanodisc densities are colored by displacement along the Z axis and the 0 Å reference height corresponds to L620 on TM10 which is in the same position in the presence and absence of Ca^{2+}. In A and B, T325 is colored in green as a visual reference point for TM4. In C-E, Y432 is colored in green as a visual reference point for TM6.

	C18/Ca^{2+}	C18/0 Ca^{2+}	C14/0 Ca^{2+}	C22/Ca^{2+} (MSP1E3)	C22/Ca^{2+} (MSP2N2)	DA/EA C14/Ca^{2+}
**Included Residues**	12-463, 487-660, 701-724	11-24,32-89, 103-255, 269–311, 318-401, 421–442, 491–593, 604–659 and 703–717	11-88, 101-400, 420-447, 489-594, 603-660, 701-728	11-260, 267-397, 419-452, 488-660, 701-727	13-260, 267-312, 317-396, 419-453, 468-660, 701-724	11-94, 102-258, 268–312, 317-398, 421–446, 489–593, 604–659 and 702–724
**Side chain truncations**	E45, K70, E121, E132, K134, R260, E262, E266, K409, E410, H411, K414, K460, E461, Y462, E488, D489, and R710	Q11,E12, D17, I33, K34, K35, E37, E42, E45, E51, D56, E57, N58, K69, K70, K73, R74, R89, E104, E106, K119, R129, E132, K134, D137, E145, E146, R149, Q150, R153, E154, S156, K157, E164, E232, S244, H247, E248, K249, E252, K254, M277, Q283, E305, F307, E310, Y319, L320, D348, E353, D356, K359, R391, D397, F399, H400, L401, N422, D424, F442, R492, E502, E504, Y506, D507, D510, D511, E514, R545, E636, R641, R642, E643, E646,	E12, D26, D29, K34, E37, E45, E57, K69, K70, H247, K254, K257, E258, E310, and R704	D28, K34, E45, E57, K100, E121, K134, E146, E232, R260, E488, R492, D489,E703, R710, and E717	K34, E37, E45, D56, E57, K69, K70, K73, Q102, K134, Y319, E488, D489, R642, E703, R710, and E714	Q11, E12, D17, N23, D26, I27, D28, T29, P30, E31, I33, K34, K35, E37, V38, L41, E42, E45, E51, D56, E57, N58, S68, K69, K70, K71, K73, R74, D83, N90, T91, E92, E94, Q102, E104, P118, H130, E132, K134, N135, D137, E145, E146, T147, R149, Q150, R153, E154, K157, E164, R168, E232, Q239, K241, H247, E248, K249, R250, E252, F253, K254, P255, E256, K257, E258, R268, V270, T274, K275,

		R647, M650, R651, K652, R653, D656, T657, E703, R704, M707, R708, R710, K713, E714, and E717.				M277, Y278, Q283, L288, L289, E305, I306, S309, E310, Y312, K317, Y319, L320, V321, I323, D348, E353, D356, Q364, N370, S390, R391, D397, V398, I421, D424, F442, L444, E445, T446, D489, E490, R492, E502, E504, D505, D507, T509, D510, M515, E543, R545, S546, D547, K550, E554, D571, N593, T604, R606, L635, E636, R641, R642, E643, E646, R647, M650, R651, K652, R653, D656, T702, E703, R704, M707, R708, R710, K713, E714, E717, L720, S721, and L722.

Supplementary Table 1: Residue ranges and side chain truncations

Supplementary Table 2: Map and model statistics for all structures.

Map Parameters	$C18/Ca^{2+}$	C18/0 Ca^{2+}	C14/0 Ca^{2+}	$C22/Ca^{2+}$ (MSP1E3)	$C22/Ca^{2+}$ (MSP2N2)	D511A/E514A $C14/Ca^{2+}$
**Final Pixel Size (Å)**	0.7066	0.85	1.06	0.7066	1.06	1.06
**Symmetry**	C2	C2	C2	C2	C2	C2
**Micrographs**	8,891	6,400	7,432	9,395	7,915	9,853
**Initial particles**	4,551,732	2,411,226	1,728,771	2,643,590	1,168,291	3,820,960
**Final particles**	994,187	151,197	244,507	132,332	31,890	155,902
**Map resolution (Å)**	2.28	3.07	3.30	2.71	3.51	3.08
**FSC threshold**	0.143	0.143	0.143	0.143	0.143	0.143
**Map local resolution range (Å)**	2.2-3.0	3.1-3.9	3.3-4.1	2.6-4.0	3.1-4.3	3.1-3.9
**Map sharpening B factor ($Å^2$)**	-71	-106.4	-118.2	-67.8	-81.7	-106.8
**Initial Model Used**	6E0H	7RX3	6DZ7	7RXG	7RXG	7RX3
**Model resolution (Å) (FSC_{model}=0.5)**	2.4	3.21	3.5	2.9	3.7	3.33
**Model Composition**						
**Nonhydrogen atoms**	11,380	8,506	10,134	10,390	9,908	8,694
**Protein residues**	1,300	1,096	1,182	1,230	1,212	1,156
**Ligands**	26	8	16	18	8	8
**r.m.s. deviations bond length (Å)**	0.005	0.009	0.007	0.009	0.008	0.005

**r.m.s. deviations bond length (Å)**	0.63	0.92	0.95	0.85	1.07	0.84
**Mean B factors ($Å^2$)**						
**Protein**	36.3	51.1	20.4	45.1	40.3	56.1
**Ligand**	24.8	43.8	21.9	54.1	N/A	53.6
**Water**	24.8	N/A	N/A	31.5	38.5	N/A
**Validation**						
**MolProbity Score**	1.42	1.14	1.85	1.52	1.95	1.23
**Clash Score**	5.43	0.72	6.88	3.47	9.12	1.32
**Poor Rotamers (%)**	0.18	0	0.2	0.19	1.35	0
**Ramachandran Plot**						
**Favored (%)**	97.36	93.96	92.46	94.38	94.61	94.57
**Allowed (%)**	2.48	6.04	7.54	5.62	5.05	5.43
**Disallowed (%)**	0.26	0	0	0	0.34	0
