# Supplementary material for: TMEM16 scramblases thin the membrane to enable lipid scrambling
Source: Nat Commun. 2022 May 11;13:2604. doi: 10.1038/s41467-022-30300-z (PMC9095706; doi:10.1038/s41467-022-30300-z)
Supplement: Supplementary file 3 — Reporting Summary [file 41467_2022_30300_MOESM3_ESM.pdf]

nature portfolio

Corresponding author(s): Alessio Accardi

Last updated by author(s): February 3rd, 2022

Reporting Summary

Nature Portfolio wishes to improve the reproducibility of the work that we publish. This form provides structure for consistency and transparency in reporting. For further information on Nature Portfolio policies, see our Editorial Policies and the Editorial Policy Checklist.

Statistics

For all statistical analyses, confirm that the following items are present in the figure legend, table legend, main text, or Methods section.

n/a	Confirmed	
☐	☒	The exact sample size (*n*) for each experimental group/condition, given as a discrete number and unit of measurement
☐	☒	A statement on whether measurements were taken from distinct samples or whether the same sample was measured repeatedly
☒	☐	The statistical test(s) used AND whether they are one- or two-sided<br>*Only common tests should be described solely by name; describe more complex techniques in the Methods section.*
☒	☐	A description of all covariates tested
☒	☐	A description of any assumptions or corrections, such as tests of normality and adjustment for multiple comparisons
☐	☒	A full description of the statistical parameters including central tendency (e.g. means) or other basic estimates (e.g. regression coefficient) AND variation (e.g. standard deviation) or associated estimates of uncertainty (e.g. confidence intervals)
☒	☐	For null hypothesis testing, the test statistic (e.g. *F*, *t*, *r*) with confidence intervals, effect sizes, degrees of freedom and *P* value noted<br>*Give P values as exact values whenever suitable.*
☒	☐	For Bayesian analysis, information on the choice of priors and Markov chain Monte Carlo settings
☒	☐	For hierarchical and complex designs, identification of the appropriate level for tests and full reporting of outcomes
☒	☐	Estimates of effect sizes (e.g. Cohen's *d*, Pearson's *r*), indicating how they were calculated

Our web collection on statistics for biologists contains articles on many of the points above.

Software and code

Policy information about availability of computer code

Data collection	CryoEM data were collected using SerialEM or Leginon.
Data analysis	CryoEM data was analyzed using relion3.0 or relion3.1_beta, CTFFIND4, and CryoSPARC v2 and cisTEM. Models were built using Coot 0.8 and refined using Phenix 1.14. Chimera 1.15, ChimerX 1.1, and Pymol 2.2 were used to visualize maps and models. SigmaPlot 10.0 and Ana were used to fit and plot functional data.

For manuscripts utilizing custom algorithms or software that are central to the research but not yet described in published literature, software must be made available to editors and reviewers. We strongly encourage code deposition in a community repository (e.g. GitHub). See the Nature Portfolio guidelines for submitting code & software for further information.

Data

Policy information about availability of data

All manuscripts must include a data availability statement. This statement should provide the following information, where applicable:

- Accession codes, unique identifiers, or web links for publicly available datasets
- A description of any restrictions on data availability
- For clinical datasets or third party data, please ensure that the statement adheres to our policy

All models and associated cryoEM maps have been deposited into the Electron Microscopy Data Bank (EMDB) and the Protein Data Bank (PDB). The depositions include final maps, unsharpened maps, and associated FSC curves. EMDB accession numbers are as follows: C18/Ca2+ dimer-EMD-24730, C18/Ca2+ monomer-EMD-24731, C22/Ca2+MSP1E3-EMD-24722, C22/Ca2+MSP2N2-EMD-24717, C18/0Ca2+-EMD-24727, C14/0Ca2+-EMD-24723, D511A/E514A C14/Ca2+-

EMD-24726. PDB accession numbers are as follows: C18/Ca2+ dimer-7RXH, C18/Ca2+ monomer-7RXG, C22/Ca2+MSP1E3-7RX2, C22/Ca2+MSP2N2-7RWJ, C18/0Ca2+-7RXB, C14/0Ca2+-7RX3, D511A/E514A C14/Ca2+-7RXA.

Field-specific reporting

Please select the one below that is the best fit for your research. If you are not sure, read the appropriate sections before making your selection.

☒ Life sciences ☐ Behavioural & social sciences ☐ Ecological, evolutionary & environmental sciences

For a reference copy of the document with all sections, see nature.com/documents/nr-reporting-summary-flat.pdf

Life sciences study design

All studies must disclose on these points even when the disclosure is negative.

Sample size	For cryoEM experiments 6400-9853 micrograph movies were recorded (exact numbers are indicated in Table 3 and in the Methods section). In all cases these numbers are sufficient to yield high resolution cryoEM structures. The number of micrograph movies used in the present study is typical for cryoEM sessions using the standard acquisition scheme and set-up described in the Methods section. Scramblase assay results represent at least three biological replicates.
Data exclusions	The sample size of the cryoEM experiments was reduced by excluding from processing unsuitable micrographs and particles using the following criteria: micrographs with low detected fit resolution estimation and those whose CTF failed to be fitted; false positive particles (i.e. ice images, contaminants and empty micelles) were excluded using 2D classification. Exact numbers of initial and final particles acquired during processing and used for 3D reconstruction for each structural experiment are indicated in Table 3 and in the respective Supp. Figure legends.<br>For the functional scrambling assay all conditions were tested side by side with a control preparation of WT afTMEM16 reconstituted in C18 lipids. In some rare cases this control sample behaved anomalously, judged by scrambling fit parameters outside 3 times the standard deviation of the mean for the wildtype. In these cases, the whole batch of experiments was disregarded.
Replication	For each cryoEM structural experiment 4-8 grids were prepared and screened. The best grid for each condition was used for cryoEM data acquisition and structural determination. The scrambling assay was performed in 6+ independent replicates from 2+ independent biological preparations. All attempts to replicate/reproduce experiments were successful
Randomization	Randomization was not relevant to the design of cryoEM experiments. Randomization was used by analysis programs for cryoEM image processing. RELION internally splits particles in two random half sets to calculate the Fourier shell correlation for validating the resolution during 3D refinement. Randomization was not relevant to the design of the functional scrambling assays as no grouping was involved in the experiments.
Blinding	The experimental design did not require blinding for structural and biochemical assays. In all cases a defined system was used.

Reporting for specific materials, systems and methods

We require information from authors about some types of materials, experimental systems and methods used in many studies. Here, indicate whether each material, system or method listed is relevant to your study. If you are not sure if a list item applies to your research, read the appropriate section before selecting a response.

Materials & experimental systems

n/a	Involved in the study	
☒	☐	Antibodies
☐	☒	Eukaryotic cell lines
☒	☐	Palaeontology and archaeology
☒	☐	Animals and other organisms
☒	☐	Human research participants
☒	☐	Clinical data
☒	☐	Dual use research of concern

Methods

n/a	Involved in the study	
☒	☐	ChIP-seq
☒	☐	Flow cytometry
☒	☐	MRI-based neuroimaging

Eukaryotic cell lines

Policy information about cell lines

Cell line source(s)	Saccharomyces cerevisiae FYG217-URA
Authentication	Cell lines were not authenticated
Mycoplasma contamination	N/A

Commonly misidentified lines<br>(See ICLAC register)	N/A
